# A Pilot Study of the CD38 Antagonist Daratumumab in Patients with Metastatic Renal Cell Carcinoma or Muscle-Invasive Bladder Cancer

**DOI:** 10.1158/2767-9764.CRC-24-0237

**Published:** 2024-09-17

**Authors:** Matthew T. Campbell, Amishi Y. Shah, Pavlos Msaouel, Nizar M. Tannir, Arlene O. Siefker-Radtke, Ashish M. Kamat, Neema Navai, Colin P.N. Dinney, Priya Rao, Charles C. Guo, Rahul A. Sheth, Aradhana M. Venkatesan, Rebecca S. Tidwell, Shalini S. Yadav, Aidi Gu, Hong Chen, Marc Macaluso, Fei Duan, Sreyashi Basu, Sonali Jindal, Padmanee Sharma

**Affiliations:** 1 Department of Genitourinary Medical Oncology, University of Texas MD Anderson Cancer Center, Houston, Texas.; 2 Department of Urology, University of Texas MD Anderson Cancer Center, Houston, Texas.; 3 Department of Pathology, University of Texas MD Anderson Cancer Center, Houston, Texas.; 4 Department of Interventional Radiology, University of Texas MD Anderson Cancer Center, Houston, Texas.; 5 Division of Diagnostic Imaging, Department of Abdominal Imaging, University of Texas MD Anderson Cancer Center, Houston, Texas.; 6 Department of Biostatistics, University of Texas MD Anderson Cancer Center, Houston, Texas.; 7 Immunotherapy Platform, University of MD Anderson Cancer Center, Houston, Texas.; 8 James P. Allison Institute, University of MD Anderson Cancer Center, Houston, Texas.; 9 Department of Immunology, University of MD Anderson Cancer Center, Houston, Texas.

## Abstract

**Purpose:**

We performed a pilot study of daratumumab (an mAb directed against CD38) in muscle-invasive bladder cancer (MIBC) and treatment-refractory metastatic renal cell carcinoma (mRCC).

**Experimental Design:**

Patients with MIBC underwent baseline transurethral resection of the bladder tumor followed by four weekly doses of daratumumab prior to cystectomy. Patients with mRCC underwent baseline and sequential biopsies after eight weekly doses. The primary endpoint was safety. The secondary endpoints were pathologic complete response rate for the MIBC cohort and objective response rate and progression-free survival for the mRCC cohort. Exploratory analyses included immune monitoring and overall survival. A Bayesian sequential monitoring design for toxicity was used for excessive toxicity.

**Results:**

In both the MIBC (*n* = 8) and mRCC (*n* = 8) cohorts, no toxicity events were encountered. In the MIBC cohort, one patient experienced pathologic complete response rate. In the mRCC cohort, no objective responses were reported, and the median progression-free survival was 1.5 months (95% confidence interval, 1.1–1.8 months). Immune monitoring found significant reductions in NK cells in circulation in both cohorts after treatment. In the tissue analysis, IHC found evidence of diminished CD38 presence in mRCC with treatment, whereas the baseline levels in MIBC were low.

**Conclusion:**

Treatment with daratumumab was safe. No signal of efficacy was detected in mRCC, and conclusions on the activity in MIBC were limited. Evidence of daratumumab targeting CD38 was detected in circulating immune cells and within the tumor microenvironment of mRCC and MIBC.

**Significance:** In this prospective clinical trial of daratumumab, treatment in patients with MIBC and mRCC was safe. Limited efficacy was observed. Treatment with daratumumab resulted in CD38-expressing immune cell subsets to be targeted both in circulation and within the tumor microenvironment.

## Introduction

The use of immune checkpoint inhibitors (ICI) revolutionized the treatment of both metastatic renal cell carcinoma (mRCC) and urothelial carcinoma. Despite the improvements in overall survival in the first-line treatment of mRCC with ICI in various combinations and in the switch maintenance or second-line setting of metastatic urothelial cancer, most patients eventually experience disease progression (1–6). Novel targets to modulate the immune-suppressive microenvironment are needed to reduce resistance to ICI therapy.

CD38 is a complex glycoprotein with ectoenzymatic activity and is highly expressed in immune cell populations including T cells, NK cells, macrophages, myeloid-derived suppressor cells, B cells, plasma cells, and dendritic cells (7, 8). In addition, CD38 serves as a critical initial enzyme in the conversion of NAD^+^ to ADP-ribose and cyclic ADPR (cADPR), leading to a downstream production of adenosine via the alternative adenosine-generating pathway creating an immune-suppressed tumor microenvironment (TME; 7, 9). Under acidic conditions, CD38 catalyzes the conversion of NAD phosphate (NADP^+^) to nicotinic acid adenine dinucleotide phosphate (NAADP^+^). Both cADPR and NAADP^+^ are regulators of calcium signaling in T-cell lymphocytes (8).

Daratumumab is an immunoglobulin G1κ human mAb targeting CD38-expressing cells. Daratumumab mediates cell death through antibody-dependent, cell-mediated cytotoxicity, antibody-mediated activation of the complement pathway, Fc-mediated cross-linking, and antibody-dependent cellular phagocytosis (10). Given the significant enrichment of CD38^+^-expressing malignant plasma cells, the initial drug development of daratumumab was in multiple myeloma. Initial studies found daratumumab to have single-agent activity in relapsed refractory multiple myeloma with an acceptable side effect profile and led to multiple phase III studies in combination with proteasome inhibitors or imides, leading to their FDA approvals (11, 12). In patients with multiple myeloma, daratumumab treatment not only led to a systemic increase in the frequency of T cells and T-cell receptor clonality but also led to a decrease in CD38-expressing immune cells, for example, regulatory T cells (Treg) expressing CD38 (10).

Upregulation of CD38 has been implicated as a mechanism of resistance to anti–programmed death receptor 1 (anti-PD1) treatment in both non–small cell lung cancer (NSCLC) and melanoma cell lines (13). In this work, the combination of anti-PD1 and anti-CD38 improved antitumoral control. In the Human Protein Atlas, CD38 protein expression in both renal cell carcinoma (RCC) and urothelial cancer is absent, but RNA levels have found low levels of expression, likely reflecting the presence of CD38-expressing immune cells.

Our group has used tissue-rich window of opportunity designs in surgically eligible patients with cisplatin-ineligible muscle-invasive bladder cancer (MIBC) and biopsy-rich studies in mRCC to explore safety, activity, and evidence of immune modulation systemically and within the TME (14, 15). With this background, we hypothesized that the delivery of daratumumab would be safe and would lead to modulation of CD38-expressing cells in the TME in the treatment of cisplatin-ineligible MIBC prior to cystectomy and in patients with mRCC previously treated with a VEGFR tyrosine kinase inhibitor (TKI) and an ICI.

## Materials and Methods

NCT03473730 was an institutional review board–approved (GU2017-0688), single-center, investigator-initiated, open-label pilot study of daratumumab monotherapy in two separate cohorts of patients: (i) surgically eligible, cisplatin-ineligible muscle-invasive urothelial cancer of the bladder (MIBC cohort) and (ii) in metastatic clear-cell RCC (mRCC cohort). Patients were required to sign written informed consent, and the study was conducted in accordance with the Declaration of Helsinki. Both sexes were eligible for enrollment. In the MIBC cohort, up to 15 patients who met study eligibility criteria received up to four weekly doses of daratumumab 16 mg/kg weekly followed by cystectomy 2 to 4 weeks after the final treatment. The primary endpoint of this cohort was safety using Common Terminology Criteria for Adverse Events version 5.0, and the number of patients who had a significant delay or were unable to undergo cystectomy as defined by at least a 4-week delay from the anticipated surgical window (> 4 weeks after the last dose of daratumumab) was evaluated. The key secondary endpoint for the MIBC cohort was pathologic complete response rate (pCR; ypT0N0). Key eligibility and ineligibility criteria included histologic confirmation of MIBC, cisplatin ineligibility as defined by the Galsky criteria (16), considered eligible for cystectomy, systemic treatment naïve (prior intravesical therapy was allowed), Eastern Cooperative Oncology Group performance status ≤2, no history of clinically significant cardiovascular disease, no evidence of acute or chronic infection, and acceptable laboratory parameters including a glomerular filtration rate as calculated using the Cockgroft–Gault formula ≥20 mL/min.

In the mRCC cohort, up to 15 patients who met study eligibility criteria would receive eight weekly doses of daratumumab 16 mg/kg followed by a biopsy or surgery and then resumption of daratumumab every other week for up to eight treatments followed by every 4-week administration. Cross-sectional imaging was performed at baseline, 8 weeks, and then every eight weeks after biopsy or surgery. The dosing was selected to mirror the approved dosing schedule in multiple myeloma outside of the tissue collection phase. Imaging could be performed earlier at the discretion of the study team. The primary endpoint for this cohort was safety using Common Terminology Criteria for Adverse Events version 5.0; the key secondary endpoints included objective response rate (ORR) using RECIST v1.1 and progression-free survival (PFS) using the Kaplan–Meier technique (17). Key eligibility and ineligibility criteria included histologic confirmation of clear cell histology and biopsy or surgery within 1 year of study enrollment, previous treatment with at least one TKI therapy and one ICI in the programmed death receptor 1 (PD1) or PDL1 class, measurable disease using RECIST v1.1 in a lesion not intended to be biopsied or undergo surgery, Eastern Cooperative Oncology Group performance status ≤2, acceptable laboratory parameters, no evidence of acute or chronic infection, and no serious cardiovascular disease.

In both cohorts, a Bayesian sequential monitoring design was used with MultiLinc Desktop version 2.1.0. employing some extensions and applications of a Bayesian strategy for monitoring multiple outcomes in clinical trials (18). Safety monitoring was continuous after the fifth patient was enrolled with strict stopping if extreme toxicity (TOX) was found at an unacceptable rate. Given the potential for cure in the MIBC cohort, the stopping rules were stricter than those in the mRCC cohort. In the MIBC cohort, the cohort was to be terminated if Prob (pTOX > 0.10 | data) >0.80 in which pTOX is the probability that a patient experienced at least one TOX event. The calculations use an *a priori* probability of toxicity following β (0.2, 1.8). Based on the stopping rules, with 15 patients in this cohort, the probability of stopping the cohort early was 11% if the true toxicity rate was 10%, only 3% if the true rate was as low as 5%, and 67% if the true rate was 30%. In the mRCC cohort, this cohort was to be terminated if Prob (pTOX > 0.30 | data) >0.925, assuming a prior of β (1,1) for pTOX. Based on these stopping rules, with 15 patients in the mRCC cohort, the probability of stopping this cohort early was 17% if the true toxicity rate was 30%, as low as 1% if the true toxicity rate was 10%, and up to 77% if the true toxicity rate was 60%. The secondary endpoints included pCR in the MIBC cohort defined as ypT0N0, with an Objective response rate (ORR) in the mRCC cohort using RECIST v1.1. The pCR and ORR rates and their 90% credibility intervals were estimated using an uninformative β distribution with a prior β (1,1). PFS was estimated by Kaplan–Meier estimates (SAS 9.4, The SAS Institute Inc.).

Patients in both cohorts provided written informed consent on an institutional review board–approved laboratory research protocol (PA13-0291). In the MIBC cohort, tissue collection occurred at baseline from the transurethral resection of the bladder tumor specimen, and posttreatment tissue was obtained from the cystectomy specimen. In the mRCC cohort, if the primary tumor was present, this was the preferred site of biopsy, and if the patient was considered eligible, they could undergo cytoreductive nephrectomy or metastasectomy. In patients not eligible for surgery, a repeat biopsy of the same location was performed.

### IHC

Representative formalin-fixed, paraffin-embedded blocks were sectioned at 4 μm. Hematoxylin and eosin staining and IHC staining were performed using CD38, CD8, CD68, and FOXP3 antibodies. Slides were scanned and digitized using the Aperio Scan Scope XT system (Leica Technologies). Quantification analysis was done using Halo software (V3.2.1851), and cell densities (cells/mm^2^) were plotted. Statistical significance was determined by the Wilcoxon paired test (untreated vs. treated). Data were analyzed using GraphPad Prism software version 8.0. *P* < 0.05 was considered statistically significant.

### Flow cytometry

Flow cytometry was performed on untreated and daratumumab-treated tissue specimens and on peripheral blood mononuclear cells at baseline. Single-cell suspensions were processed for flow cytometry assay. Expression of the following markers was assessed: CD45, CD3, CD4, CD8, CD127, CD25, CD19, CD11b, CD14, HLA-DR, FOXP3, CD56, CD38, and CSF-1R. Flow analysis was performed using FlowJo v10 (BD Biosciences). Data were analyzed using GraphPad Prism software version 8.0. Statistical significance was determined by the Mann–Whitney test for tissue and peripheral blood mononuclear cell samples. *P* < 0.05 was considered statistically significant.

### NanoString transcriptional profiling

Stored cells from tissue specimens were processed with RiboPure RNA Purification Kit (Thermo Fisher Scientific) according to the manufacturer’s protocol. Extracted RNA was quantified using a NanoDrop 1000 spectrometer (Thermo Fisher Scientific). For NanoString assay, 100 ng of RNA was used to detect immune gene expression using an nCounter PanCancer Immune Profiling panel along with custom CodeSets. Counts of the reporter probes were tabulated for each sample using the nCounter Digital Analyzer, and the raw data output were imported into nSolver (http://www.nanostring.com/products/nSolver, v4.0). The nSolver data analysis package was used for normalization and cell-type analysis. Data were plotted using GraphPad Prism 9 (GraphPad Software v-9.5.1). Statistical analysis was performed using the Wilcoxon matched-pairs signed rank test for comparing paired samples and Mann–Whitney test for unpaired samples. *P* values ≤0.05 denote significant differences.

### Data availability

All raw source data used to generate the figures in the article and in the supplementary section are available upon request to the corresponding author. The Human Protein Atlas reference is www.proteinatlas.org.

## Results

### Baseline demographics

The baseline demographics for both cohorts are shown in Table 1. In the MIBC cohort, the median age was 75 years (range, 67–86 years); the majority of patients were male (88%) and White non-Hispanic (88%), and 50% had cT2, whereas 50% had cT3 at baseline. All patients were cisplatin ineligible with 50% having renal insufficiency, 25% with hearing loss and renal insufficiency, 13% with hearing loss only, and 13% with peripheral neuropathy. The median glomerular filtration rate was 44 mL (about 1.49 oz)/minutes (range, 34–138 mL/min). Using MD Anderson’s risk criteria for relapse after cystectomy, 75% had a high-risk feature with 38% having hydronephrosis, 13% having a micropapillary histology component, 13% having a positive mass on examination under anesthesia, and 13% with lymphovascular invasion (19). Finally, two patients had received prior Bacillus Calmette–Guerin treatment. In the mRCC cohort, the median age was 57 years (range, 41–73 years), and most patients were male (75%) and White non-Hispanic (75%). Using the International Metastatic Renal Cell Carcinoma Database Consortium scoring, 13% had favorable risk, 75% had intermediate risk, and 13% had poor risk disease (20). Patients were heavily pretreated with a minimum of two prior treatment lines including at least one antiangiogenic TKI and with PD1 or PDL1 agent, with 88% of participants having prior progression on cabozantinib. In terms of metastatic sites, 38% had liver metastases, 38% had bone metastases, and 25% had previously treated brain metastases. The consort diagram for trial participants is shown in Supplementary Fig. S1. Supplementary Table S1 includes the patient representativeness table.

**Table 1:** Baseline patient and tumor characteristics by disease cohort

Patient characteristic	Bladder *N* (%)	Renal *N* (%)
All	8 (100%)	8 (100%)
Age (mean, range in years)	75 (67–86)	58 (41–73)
Race/ethnicity		
Any race/Hispanic	1 (13%)	2 (25%)
White/non-Hispanic	7 (88%)	6 (75%)
Gender		
Female	1 (13%)	2 (25%)
Male	7 (88%)	6 (75%)
ECOG PS		
0	2 (25%)	5 (63%)
1	6 (75%)	2 (25%)
2	0 (0%)	1 (13%)
Cisplatin ineligibility reason		
Hearing	1 (13%)	
Neuropathy	1 (13%)	
Renal function	4 (50%)	
Renal function and hearing	2 (25%)	
Clinical stage		
T2N0M0	4 (50%)	
T3aN0M0	3 (38%)	
T3bN0M0	1 (13%)	
Prior BCG	2 (25%)	
Variant histology	3 (38%)	
MDACC high-risk algorithm		
High-risk variant (MDACC)	1 (13%)	
Hydronephrosis	3 (38%)	
Lymphovascular invasion	1 (13%)	
Palpable 3D mass EUA	1 (13%)	
None	2 (25%)	
IMDC score		
Favorable		1 (13%)
Intermediate		6 (75%)
Poor		1 (13%)
Prior PD1/PDL1		8 (100%)
Prior TKI		8 (100%)
Prior cabozantinib		7 (88%)
History of nephrectomy		7 (88%)
Liver metastases		3 (38%)
Bone metastases		3 (38%)
Brain metastases		2 (25%)

Abbreviations: BCG, Bacillus Calmette–Guerin; ECOG PS, Eastern Cooperative Oncology Group performance status; EUA, Examination Under Anesthesia; IMDC, International Metastatic Renal Cell Carcinoma Database Consortium; MDACC, MD Anderson Cancer Center.

### Safety

Tables 2 and 3 show the treatment-related toxicities encountered in the study. In the MIBC cohort, daratumumab was well tolerated with only grade 1 and 2 treatment-related toxicities. The most common treatment-related toxicity was infusion reactions in 38% of participants. One participant did not undergo surgery because of disease progression, and one patient withdrew consent after receipt of four doses of daratumumab declining surgery. In the mRCC cohort, most of the treatment-related adverse events were of grades 1 and 2. Attributed grade 3 events occurred in one patient who experienced fatigue and muscle cramps. The most common grade 2 event was infusion reactions which occurred in 25% of the participants. No TOX events occurred during the conduct of the study, and no new safety signal was generated. All adverse events in the mRCC and MIBC cohorts are included in Supplementary Tables S2 and S3, respectively. The study was terminated early because of slow accrual due to competing studies and the COVID-19 pandemic.

**Table 2:** Treatment-related adverse events in the mRCC cohort

Adverse event (*n*)	Grade			Total
	1	2	3	
Fatigue	3	1	1	5
Infusion-related reaction	0	2	0	2
Dyspnea	2	0	0	2
Cough	1	1	0	2
Fever	2	0	0	2
Sweating	1	0	0	1
Edema limbs	1	0	0	1
Muscle cramps	0	0	1	1
Chills	1	0	0	1
Pain	0	1	0	1
Hyperkalemia	0	1	0	1
Urticaria	0	1	0	1
Alanine aminotransferase increased	1	0	0	1
Chest tightness	1	0	0	1
Headache	1	0	0	1
Vomiting	1	0	0	1

**Table 3:** Treatment-related adverse events in the MIBC cohort

Adverse event (*n*)	Grade		Total
	1	2	
Infusion-related reaction	0	3	3
Pain	1	0	1
Fatigue	1	0	1
Rash	1	0	1
Nausea	1	0	1
Weight loss	1	0	1
Edema limbs	1	0	1
Constipation	1	0	1
Pruritus	1	0	1
Flushing	1	0	1
Sore throat	1	0	1
Vomiting	0	1	1
Hematuria	1	0	1
Alanine aminotransferase increased	1	0	1
Aspartate aminotransferase increased	1	0	1
Diarrhea	1	0	1
Headache	0	1	1
Urticaria	0	1	1

### Efficacy

Table 4 shows the efficacy outcomes for both the MIBC and renal cohorts. In the MIBC cohort, all eight patients completed the four planned doses of daratumumab. One patient had disease progression prior to surgery, and one patient withdrew consent prior to surgery. Of the six patients who underwent surgery, one patient had ypT0N0 disease, one patient had ypTcisN0 disease, two patients had organ-confined disease (ypT3aN0), and two patients had progression to lymph node–positive disease (ypT3bN1 and ypT3bN2, respectively). The posterior pCR rate was 20% (4%, 43%). No significant postoperative complications were encountered. Supplementary Fig. S2A and S2B shows the Kaplan–Meier curves for OS and PFS, respectively.

**Table 4:** Efficacy outcomes for the MIBC and mRCC cohorts

MIBC cohort (*N* = 8)	
>4-week surgery delay–*N*	0
ypT0N0–*N* (%) empirical	1 (12.5%)
<ypT2N0–*N* (%) empirical	2 (25%)
ypT0N0–posterior estimate (90% credible interval)	20% (4%, 43%)
<ypT2N0–posterior estimate (90% credible interval)	30% (10%, 55%)
OS median months (95% CI)	24.5 (11.2, NR)
RFS median months (95% CI)	17.7 (7.6, 19.4)
mRCC cohort (*N* = 8)	
ORR–*N* (%) empirical	0 (0%)
ORR–posterior estimate (90% credible interval)	10% (1%, 28%)
OS median months (95% CI)	9.1 (1.9, NR)
PFS median months (95% CI)	1.5 (1.1, 1.8)

Posterior estimates assume a prior β (1,1).

Abbreviations: CI, confidence interval; NR, not reached; OS, overall survival; RFS, relapse-free survival.

Median follow-up time is 21.1 months for the MIBC cohort and 27.6 months for the mRCC cohort.

In the mRCC cohort, only two patients completed the planned eight doses of weekly daratumumab. The median number of doses was seven (range, 4–15). One patient had stable disease as the best response, whereas all remaining seven patients had progressive disease as the best response. All patients stopped treatment because of disease progression. The median progression-free interval was 1.5 months (95% CI, 1.1–1.8 months). Supplementary Fig. S2 shows the Kaplan–Meier curve estimates for overall survival and relapse-free survival in MIBC, and Supplementary Fig. S3A and S3B shows Kaplan–Meier curves for OS and PFS, respectively, in patients in the renal cohort.

### Correlative analysis

**Figure 1:**
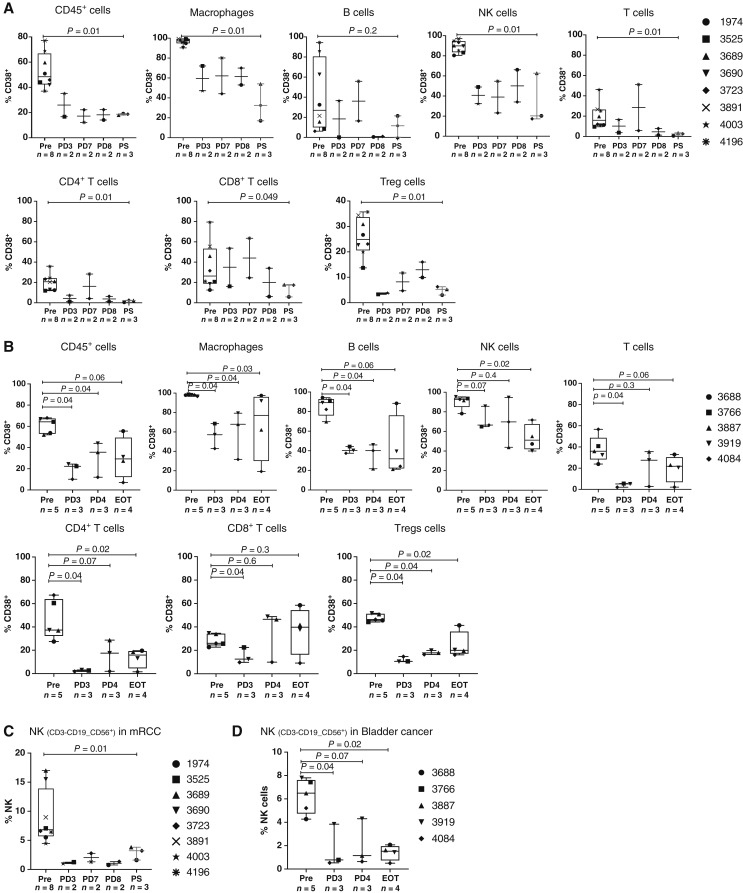
Flow cytometry of CD38^+^ immune cells from serially collected blood samples. **A,** mRCC cohort. **B,** MIBC cohort. **C,** NK cells in the mRCC cohort. **D,** NK cells in the MIBC cohort. EOT, end of treatment; PD3, predose 3; PD4, predose 4; PD7, predose 7; PD8, predose 8; Pre, pretreatment; PS, post–tissue collection.

In multiple myeloma, daratumumab’s pharmacodynamic activity was observed in circulation in which a significant decrease in CD38 expression was observed on immune cells (10). Similarly, to evaluate target engagement, we performed flow cytometry on pre- and posttreatment longitudinal blood samples obtained from the MIBC cohort. Five patients had baseline blood collected, three had a predose blood draw prior to the third dose, three had a predose blood draw prior to the fourth dose, and four had a postsurgical blood draw at the end of treatment postsurgical visit. Daratumumab treatment led to a sustained and significant decrease in the frequency of CD38^+^ immune cells, including CD45^+^ cells, macrophages, B cells, NK cells, T cells, CD4 T cells, CD8 T cells, and Tregs in both patients with mRCC and MIBC (Fig. 1A and B, respectively). We did not observe any consistent differences in circulating immune cells upon treatment with daratumumab except NK cells, a known PD marker for daratumumab treatment, which showed a significant decrease in frequency after daratumumab treatment in both tumor types in both tissues (Fig. 1C for mRCC and Fig. 1D for MIBC). Flow cytometry in immune cell populations in serially collected peripheral blood samples is shown in Supplementary Fig. S4A and S4B for the mRCC and MIBC cohorts, respectively, with significant changes observed in the NK-cell populations.

**Figure 2:**
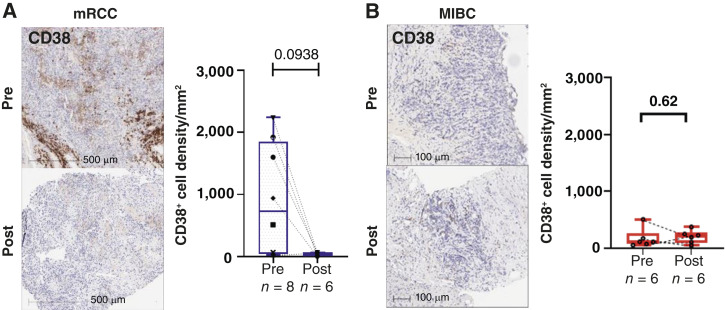
IHC for CD38 expression before and after daratumumab treatment in cohorts: **A,** mRCC. **B,** MIBC. Post, after daratumumab treatment; Pre, before daratumumab treatment.

**Figure 3:**
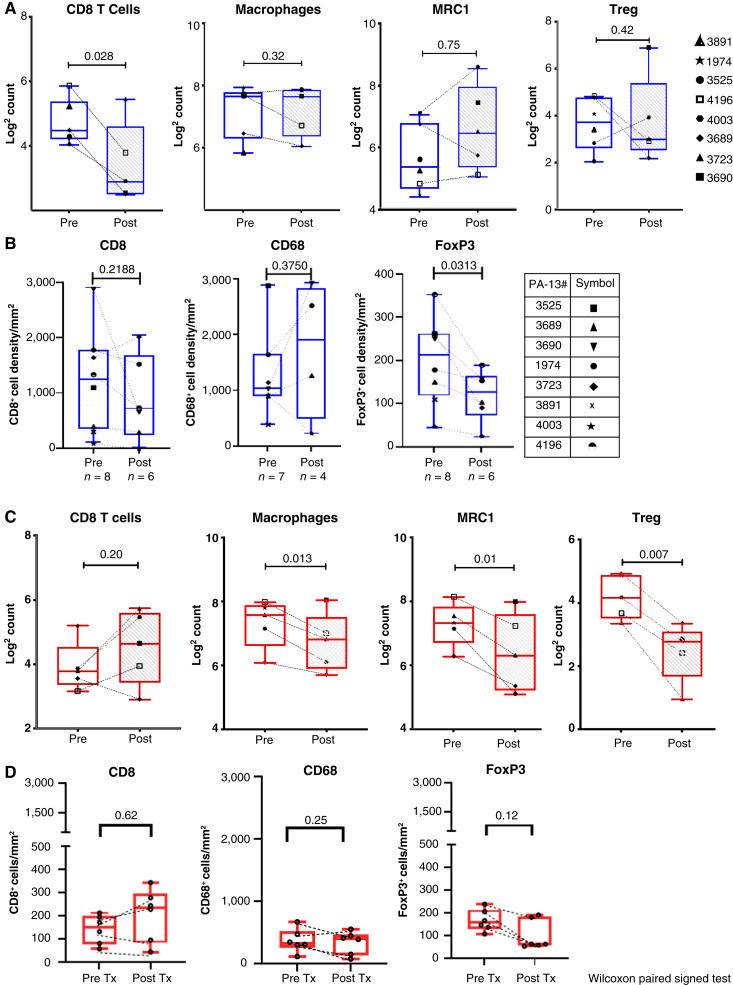
TME evolution of gene expression and IHC before and after treatment with daratumumab. **A,** mRCC NanoString. **B,** mRCC IHC. **C,** MIBC NanoString. **D,** MIBC IHC. Post, after daratumumab treatment; Pre, before daratumumab treatment; Tx, treatment.

We evaluated pre– and post–daratumumab-treated tumor tissue samples for target engagement within the TME using IHC. In mRCC tumor tissue, a trend of decreased density of CD38-expressing cells was observed with post–daratumumab treatment (Fig. 2A). Surprisingly, we did not observe any difference in CD38 expression in the MIBC cohort when we compared pre- versus posttreatment (cystectomy) specimens (Fig. 2B). In addition, the MIBC cohort pretreatment tissue samples had a lower density of CD38-expressing cells (53–505 cell density/mm^2^) compared with mRCC pretreatment tissue samples (25–2,240 cell density/mm^2^). Gene expression analysis of pre- and posttreatment tissues showed no changes in immune cell scores for CD8 T cells, macrophages, Tregs, and expression of M2-like macrophage marker CD206 (MRC1) in the mRCC cohort (Fig. 3A). In contrast, in the MIBC cohort, there was a significant decrease in macrophages and Tregs and expression of CD206, suggesting attenuation of the immunosuppressive TME in bladder cancer upon daratumumab treatment (Fig. 3C). Furthermore, IHC analysis for immune cell markers CD8, CD68, and FOXP3 in mRCC and MIBC showed similar trends as the gene expression data (Fig. 3B and D).

**Figure 4:**
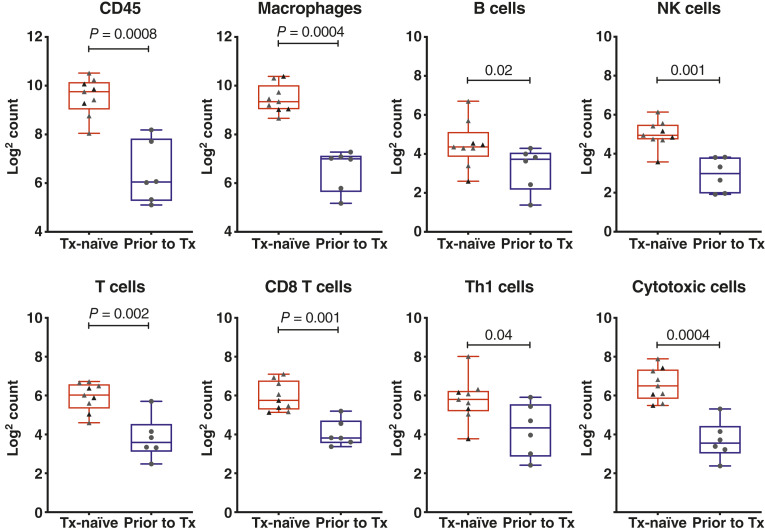
Gene expression analysis comparing immune infiltration in the mRCC treatment-naïve cohort and mRCC cohort from the daratumumab study (prior to treatment) and showing significant reduction across immune cell subtypes in heavily pretreated patients. Tx, treatment.

In the mRCC cohort, our group sought to explore why despite evidence of target engagement in the TME with CD38^+^-expressing cells, important immune cell subsets were not changed. We hypothesized in the setting of prior to exposure to antiangiogenic TKIs and prior ICT, these patients may possess an immunosuppressed TME. To test this, we performed a daratumumab *post hoc* comparison of treatment-naïve patient tissue samples from mRCC enrolled in a separate protocol NCT02626130 with pretreatment samples obtained from this heavily pretreated cohort. Gene expression analysis found that compared with the treatment-naïve cohort, the heavily pretreated cohort had significantly lower immune cell infiltration including CD45, macrophages, B, NK, T, CD8 T, aTh1, and cytotoxic cells (Fig. 4).

## Discussion

The role of targeting CD38-expressing cells in the treatment of solid malignancies has not been reported (8). In the treatment of multiple myeloma, the malignant cell expresses CD38, and therapeutic targeting with daratumumab has single-agent activity and improved outcomes in multiple combinations (11, 12). CD38 is known to be expressed on subsets of innate immune cells including macrophages and NK cells and in subsets of the adaptive immune system including B cells, plasma cells, and T cells. In the treatment of multiple myeloma, daratumumab was reported to reduce circulating CD38^+^ NK cells in a dose-dependent manner (21). In our study, we sought to explore the role of targeting CD38^+^ cells both in the circulation and in tumoral tissue in the treatment of MIBC and mRCC.

Two distinct cohorts of patients were enrolled with systemic treatment–naïve, cisplatin-ineligible patients with MIBC and heavily pretreated patients with metastatic clear-cell RCC. The rationale for exploring cisplatin-ineligible MIBC was due to a lack of available systemic treatment options for this subset. A window of opportunity was used to provide 4 weeks of daratumumab which would not delay a curative intent cystectomy that was performed 6 to 8 weeks after trial enrollment. The heavily pretreated subset of mRCC was selected because of lack of systemic treatment options and our hypothesis that a substantially immune-suppressed microenvironment could be altered with the targeting of CD38-expressing cells in the immune microenvironment.

Daratumumab administration yielded no new safety signals in either cohort. No evidence of single-agent efficacy was generated with daratumumab in heavily pretreated mRCC. Based on these findings, we do not recommend further exploration of single-agent daratumumab in mRCC. Although two patients who received daratumumab prior to cystectomy had no evidence of invasive urothelial cancer at the time of cystectomy, given the limited sample size, no determination of single-agent efficacy could be ascertained.

The correlative analysis found evidence that daratumumab effectively targeted CD38-expressing immune cells in circulation. The tissue studies in both MIBC and mRCC found low baseline expression of CD38 which was not altered with the treatment of daratumumab. Favorable effects on the bladder TME were found with a reduction of CD206-expressing macrophages and Tregs after treatment. In the mRCC cohort, IHC found reduced CD38-expressing cells after daratumumab treatment providing evidence that daratumumab can target the TME in solid tumors.

The role of combination CD38 blockade with anti-PD1 therapy has been explored in the treatment of multiple myeloma and in a phase I/II study of metastatic castrate-resistant prostate cancer and NSCLC (22, 23). In multiple myeloma, the decision was made to halt two studies with pembrolizumab in combination with either lenalidomide plus dexamethasone or pomalidomide plus dexamethasone after interim analysis raised safety concerns for excessive risk of serious adverse events including death. This decision led to the halting of multiple combination studies with anti-PD1 therapy including studies testing the combination with daratumumab (24, 25). In metastatic castrate-resistant prostate cancer and NSCLC, a study of isatuximab plus cemiplimab resulted in an acceptable safety profile but limited clinical activity.

Our study had several limitations that are important to highlight. The study stopped early because of slow accrual that was impacted by the COVID-19 pandemic and competing studies. In addition, adjusting for multiplicity in the correlative analyses was not performed given the small sample sizes involved. Therefore, definitive conclusions on the efficacy and the correlative analysis are not possible with all findings being hypothesis-generating only.

## Conclusions

In this small, phase II study, daratumumab treatment for cisplatin-ineligible MIBC and mRCC was safe. The study was terminated early because of slow accrual impacted by competing studies and the COVID-19 pandemic. The efficacy of single-agent targeting of CD38-expressing cells was not detected in mRCC, and the MIBC cohort was too small to estimate activity. Daratumumab was found to diminish CD38-expressing cells in circulation and in mRCC tissue–based analysis.

## Supplementary Material

Supplementary Table 1 Patient Representativeness Table

Supplementary Table 2 All Adverse Events in the mRCC cohort regardless of attribution

Supplementary Table 3 All Adverse Events in the MIBC cohort regardless of attribution

Supplementary Figure 1 Consort Diagram for participants in study

Supplementary Figure 2 Kaplan Meier curves in MIBC cohort A) Overall Survival and B) Relapse Free Survival (RFS)

Supplementary Figure 3 Kaplan Meier curves in renal cohort A) Overall Survival and B) Progression Free Survival (PFS)

Supplementary Figure 4 Flow cytometry of immune cells from serially collected blood samples with evidence of significant decrease in circulating NK cells in: A) Metastatic renal cell carcinoma cohort. B) MIBC cohort.
