# Supplementary material for: A Pilot Study of the CD38 Antagonist Daratumumab in Patients with Metastatic Renal Cell Carcinoma or Muscle-Invasive Bladder Cancer
Source: Cancer Res Commun. 2024 Sep 17;4(9):2444–53. doi: 10.1158/2767-9764.CRC-24-0237 (PMC11406637; doi:10.1158/2767-9764.CRC-24-0237)
Supplement: Supplementary Table 1 — Patient Representativeness Table [file crc-24-0237_supplementary_table_1_suppst1.pptx]

## Slide 1
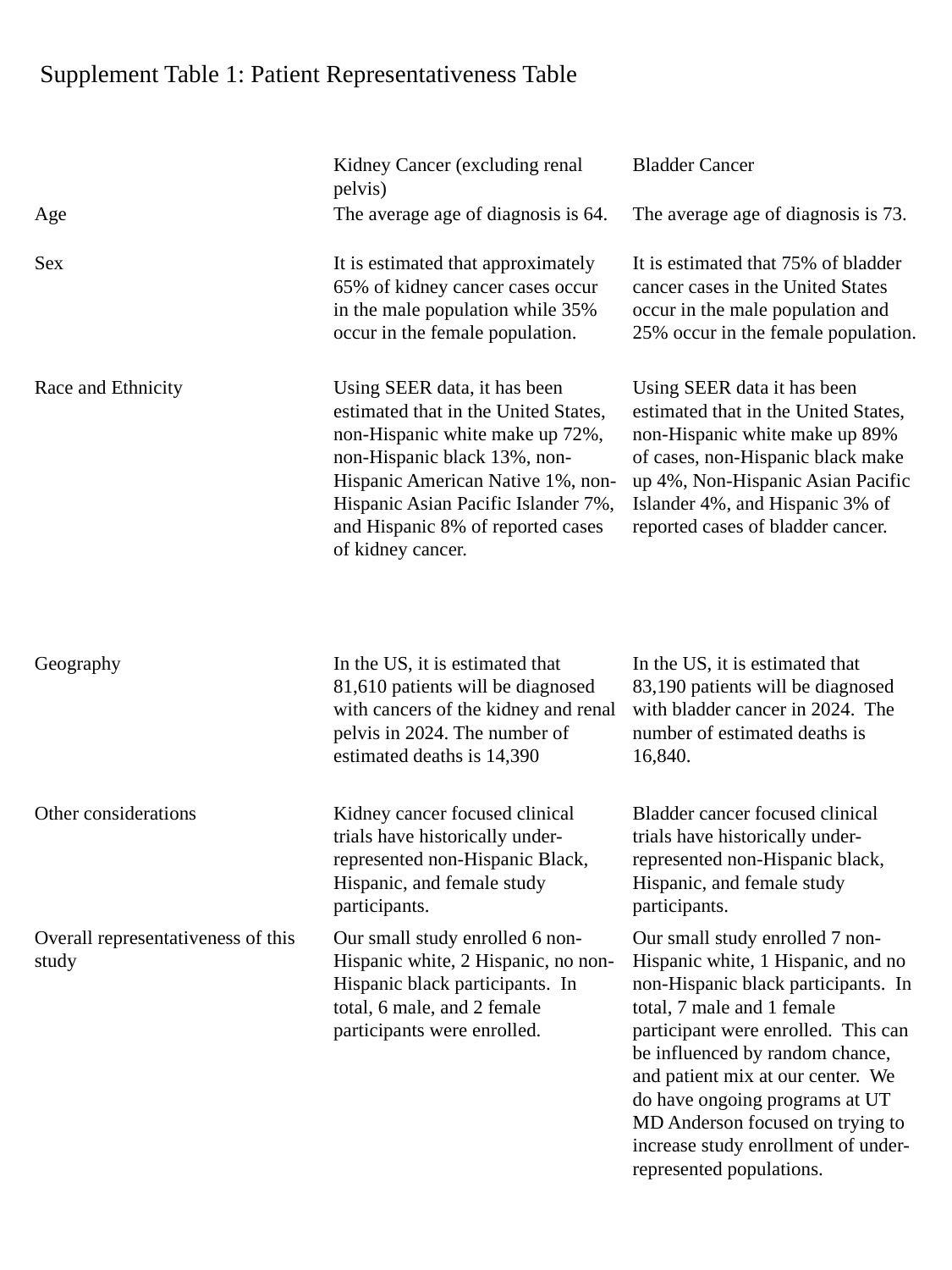

# Supplement Table 1: Patient Representativeness Table
| | Kidney Cancer (excluding renal pelvis) | Bladder Cancer |
| --- | --- | --- |
| Age | The average age of diagnosis is 64. | The average age of diagnosis is 73. |
| Sex | It is estimated that approximately 65% of kidney cancer cases occur in the male population while 35% occur in the female population. | It is estimated that 75% of bladder cancer cases in the United States occur in the male population and 25% occur in the female population. |
| Race and Ethnicity | Using SEER data, it has been estimated that in the United States, non-Hispanic white make up 72%, non-Hispanic black 13%, non-Hispanic American Native 1%, non-Hispanic Asian Pacific Islander 7%, and Hispanic 8% of reported cases of kidney cancer. | Using SEER data it has been estimated that in the United States, non-Hispanic white make up 89% of cases, non-Hispanic black make up 4%, Non-Hispanic Asian Pacific Islander 4%, and Hispanic 3% of reported cases of bladder cancer. |
| Geography | In the US, it is estimated that 81,610 patients will be diagnosed with cancers of the kidney and renal pelvis in 2024. The number of estimated deaths is 14,390 | In the US, it is estimated that 83,190 patients will be diagnosed with bladder cancer in 2024. The number of estimated deaths is 16,840. |
| Other considerations | Kidney cancer focused clinical trials have historically under-represented non-Hispanic Black, Hispanic, and female study participants. | Bladder cancer focused clinical trials have historically under-represented non-Hispanic black, Hispanic, and female study participants. |
| Overall representativeness of this study | Our small study enrolled 6 non-Hispanic white, 2 Hispanic, no non-Hispanic black participants. In total, 6 male, and 2 female participants were enrolled. | Our small study enrolled 7 non-Hispanic white, 1 Hispanic, and no non-Hispanic black participants. In total, 7 male and 1 female participant were enrolled. This can be influenced by random chance, and patient mix at our center. We do have ongoing programs at UT MD Anderson focused on trying to increase study enrollment of under-represented populations. |
