# Supplementary material for: A Pilot Study of the CD38 Antagonist Daratumumab in Patients with Metastatic Renal Cell Carcinoma or Muscle-Invasive Bladder Cancer
Source: Cancer Res Commun. 2024 Sep 17;4(9):2444–53. doi: 10.1158/2767-9764.CRC-24-0237 (PMC11406637; doi:10.1158/2767-9764.CRC-24-0237)
Supplement: Supplementary Table 3 — All Adverse Events in the MIBC cohort regardless of attribution [file crc-24-0237_supplementary_table_3_suppst3.pptx]

## Slide 1
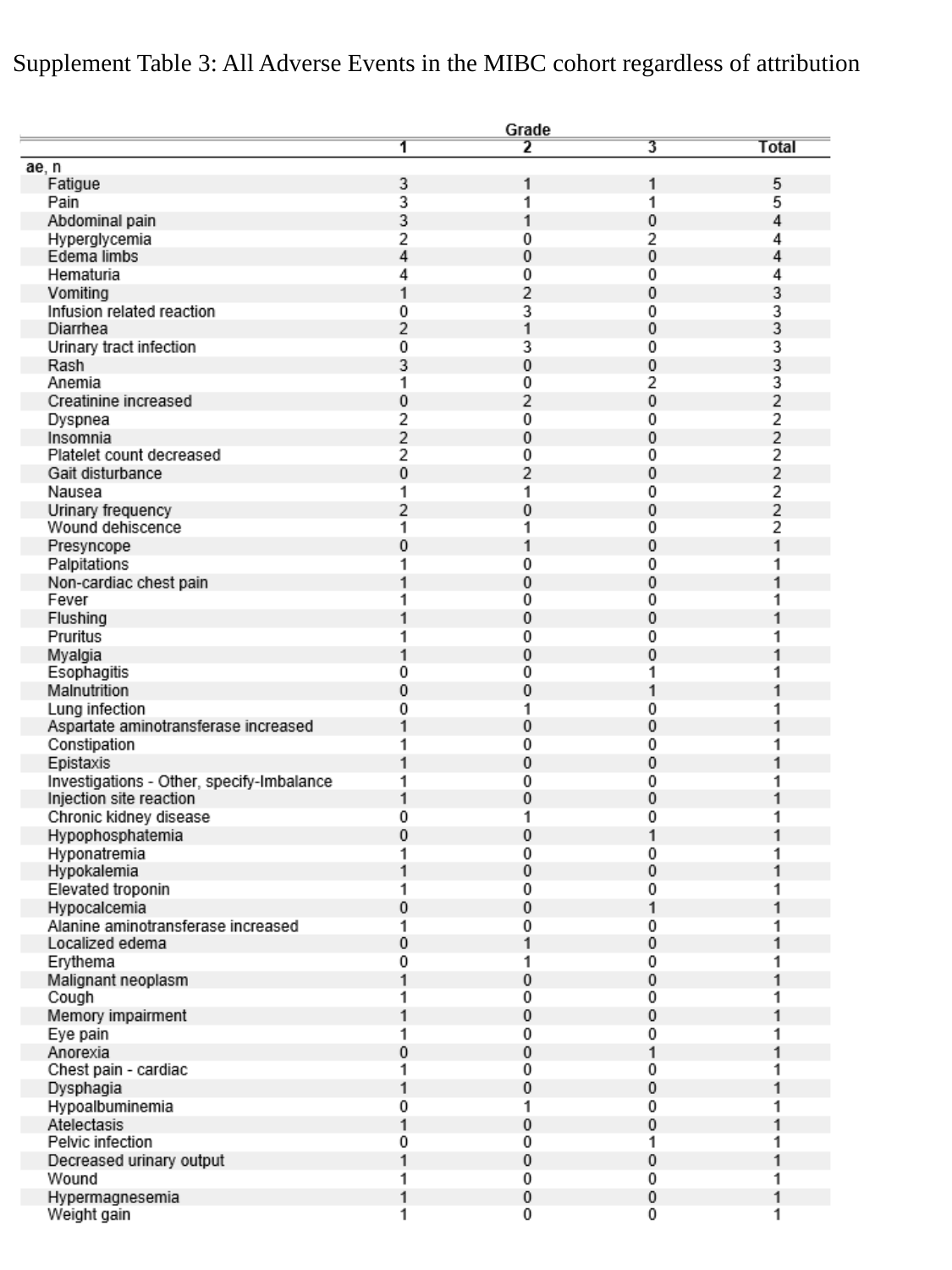

# Supplement Table 3: All Adverse Events in the MIBC cohort regardless of attribution

## Slide 2
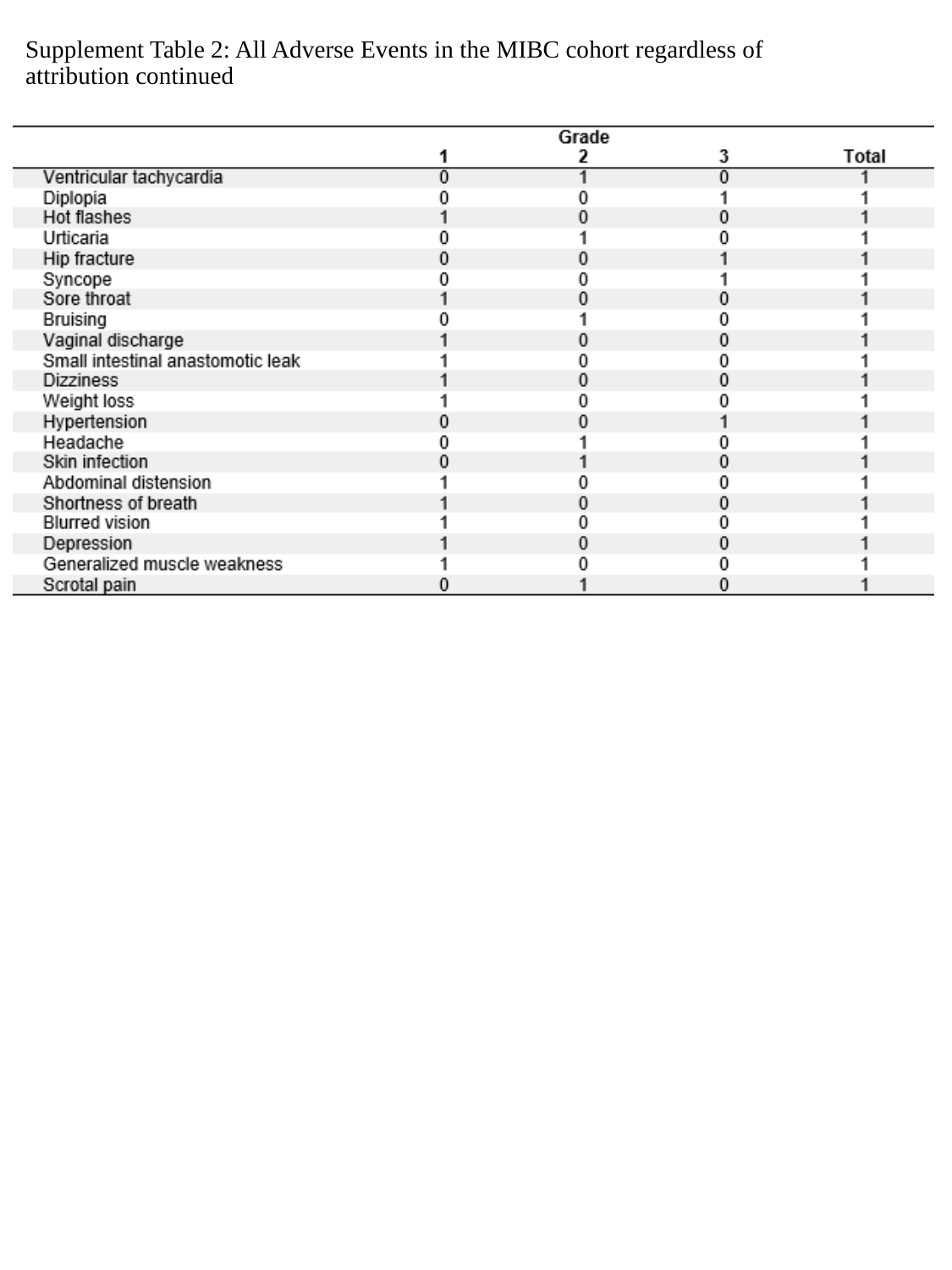

# Supplement Table 2: All Adverse Events in the MIBC cohort regardless of attribution continued
