# Supplementary material for: A Pilot Study of the CD38 Antagonist Daratumumab in Patients with Metastatic Renal Cell Carcinoma or Muscle-Invasive Bladder Cancer
Source: Cancer Res Commun. 2024 Sep 17;4(9):2444–53. doi: 10.1158/2767-9764.CRC-24-0237 (PMC11406637; doi:10.1158/2767-9764.CRC-24-0237)
Supplement: Supplementary Figure 1 — Consort Diagram for participants in study [file crc-24-0237_supplementary_figure_1_suppsf1.pptx]

## Slide 1
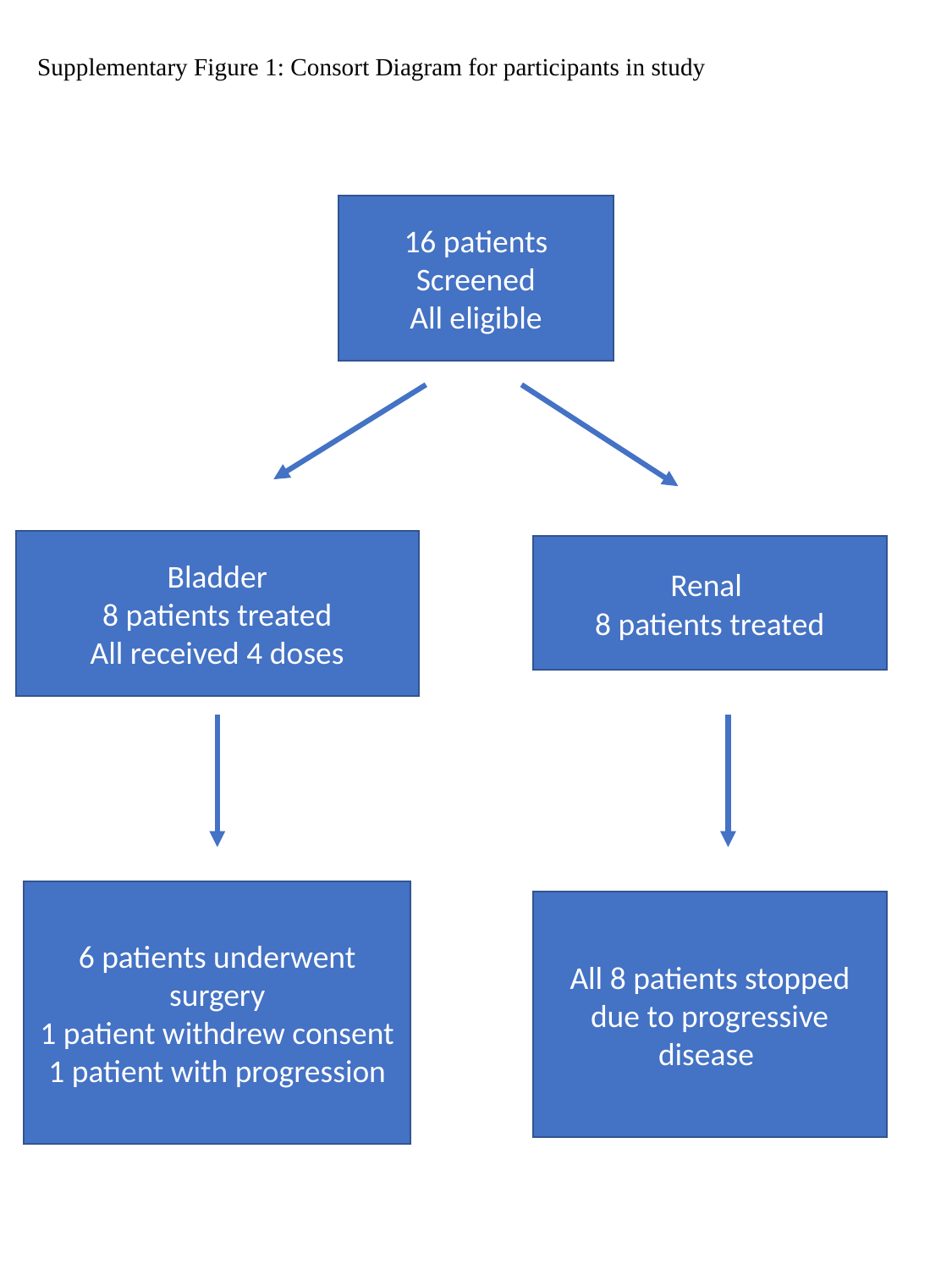

Supplementary Figure 1: Consort Diagram for participants in study
#
16 patients
Screened
All eligible
Bladder
8 patients treated
All received 4 doses
Renal
8 patients treated
6 patients underwent surgery
1 patient withdrew consent
1 patient with progression
All 8 patients stopped due to progressive disease
