# Supplementary material for: A Pilot Study of the CD38 Antagonist Daratumumab in Patients with Metastatic Renal Cell Carcinoma or Muscle-Invasive Bladder Cancer
Source: Cancer Res Commun. 2024 Sep 17;4(9):2444–53. doi: 10.1158/2767-9764.CRC-24-0237 (PMC11406637; doi:10.1158/2767-9764.CRC-24-0237)
Supplement: Supplementary Figure 2 — Kaplan Meier curves in MIBC cohort A) Overall Survival and B) Relapse Free Survival (RFS) [file crc-24-0237_supplementary_figure_2_suppsf2.pptx]

## Slide 1
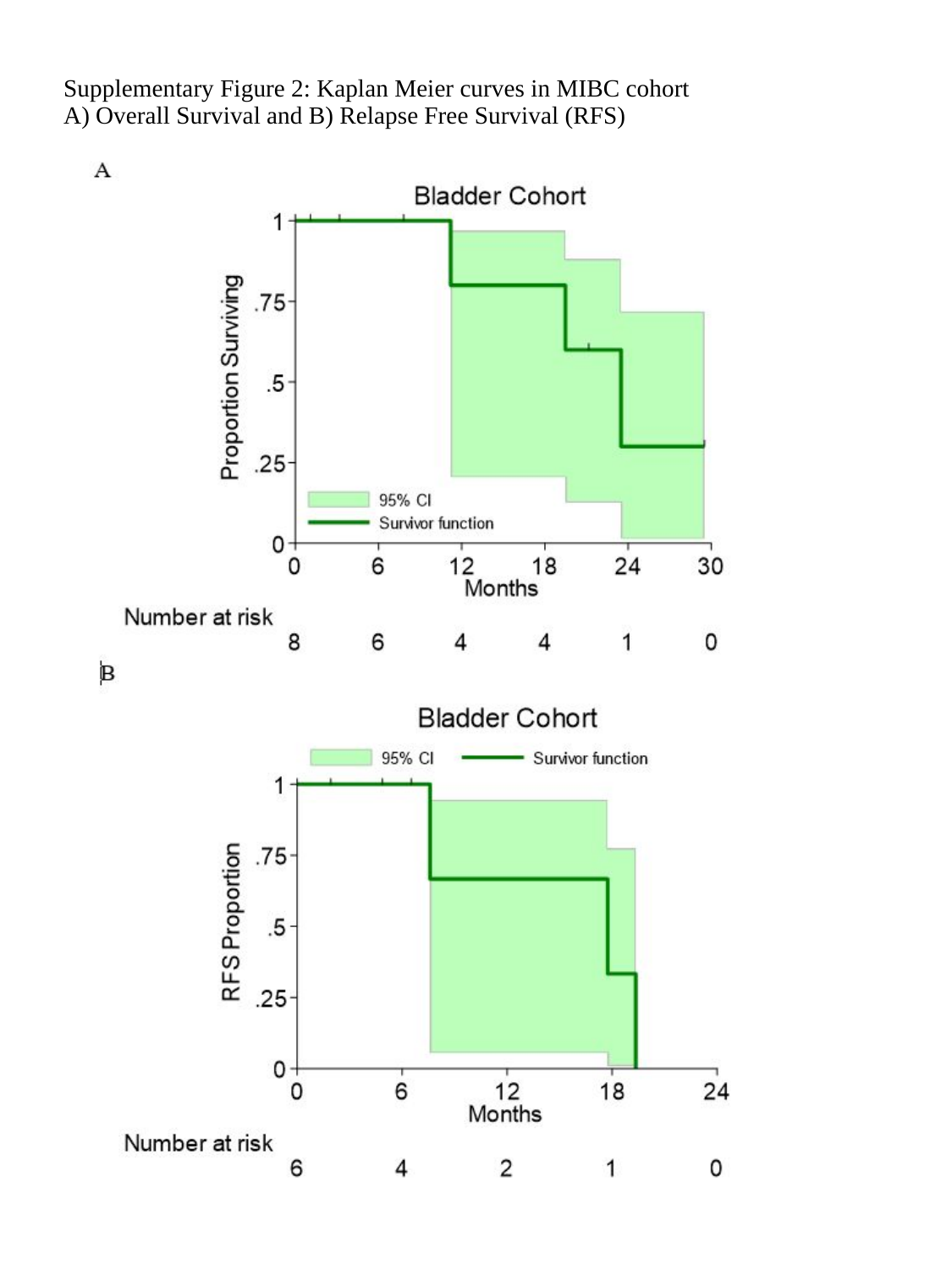

# Supplementary Figure 2: Kaplan Meier curves in MIBC cohort A) Overall Survival and B) Relapse Free Survival (RFS)
