# Supplementary material for: A Pilot Study of the CD38 Antagonist Daratumumab in Patients with Metastatic Renal Cell Carcinoma or Muscle-Invasive Bladder Cancer
Source: Cancer Res Commun. 2024 Sep 17;4(9):2444–53. doi: 10.1158/2767-9764.CRC-24-0237 (PMC11406637; doi:10.1158/2767-9764.CRC-24-0237)
Supplement: Supplementary Figure 3 — Kaplan Meier curves in renal cohort A) Overall Survival and B) Progression Free Survival (PFS) [file crc-24-0237_supplementary_figure_3_suppsf3.pptx]

## Slide 1
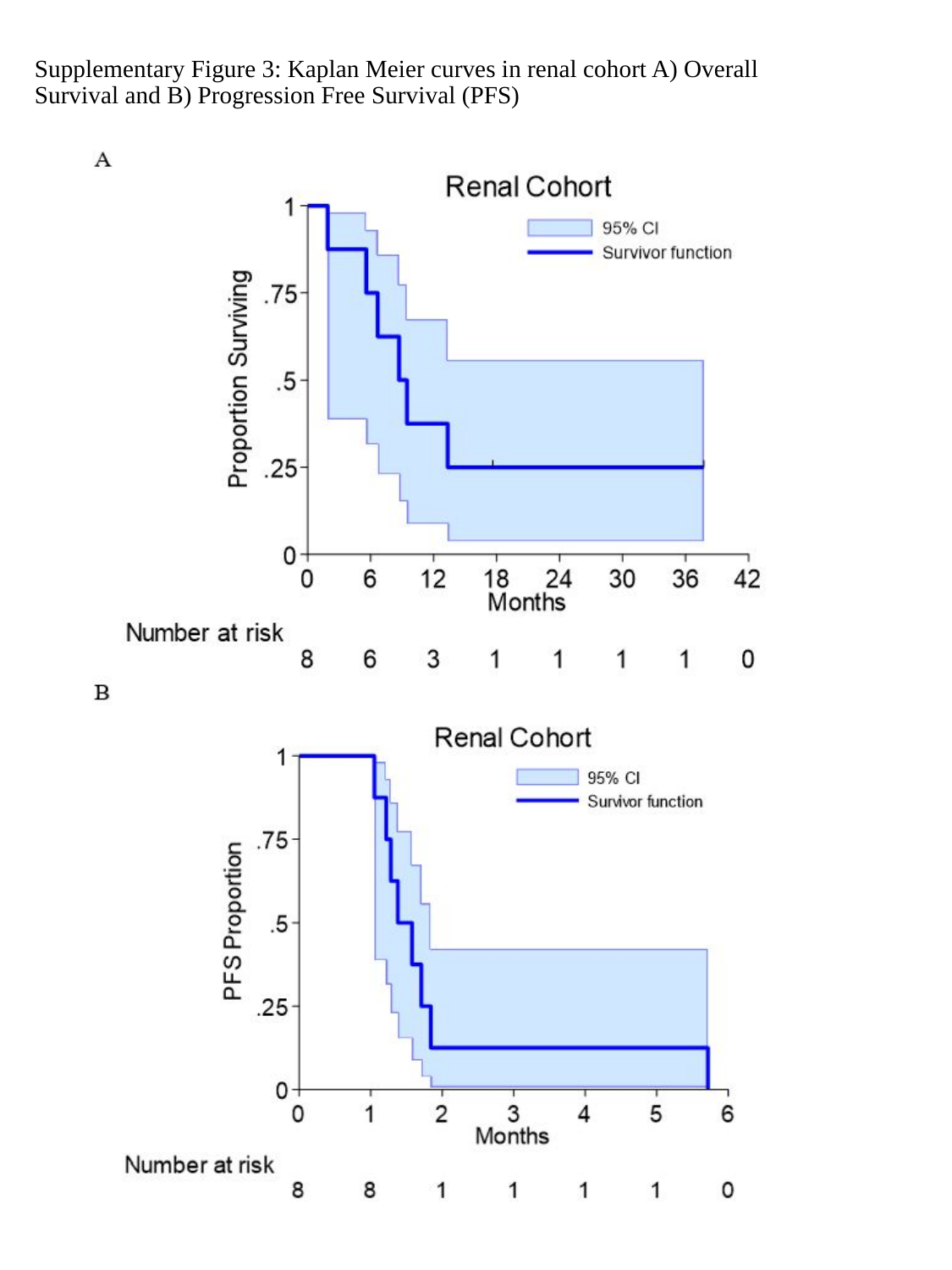

# Supplementary Figure 3: Kaplan Meier curves in renal cohort A) Overall Survival and B) Progression Free Survival (PFS)
