# Supplementary material for: A Pilot Study of the CD38 Antagonist Daratumumab in Patients with Metastatic Renal Cell Carcinoma or Muscle-Invasive Bladder Cancer
Source: Cancer Res Commun. 2024 Sep 17;4(9):2444–53. doi: 10.1158/2767-9764.CRC-24-0237 (PMC11406637; doi:10.1158/2767-9764.CRC-24-0237)
Supplement: Supplementary Figure 4 — Flow cytometry of immune cells from serially collected blood samples with evidence of significant decrease in circulating NK cells in: A) Metastatic renal cell carcinoma cohort. B) MIBC cohort. [file crc-24-0237_supplementary_figure_4_suppsf4.pptx]

## Slide 1
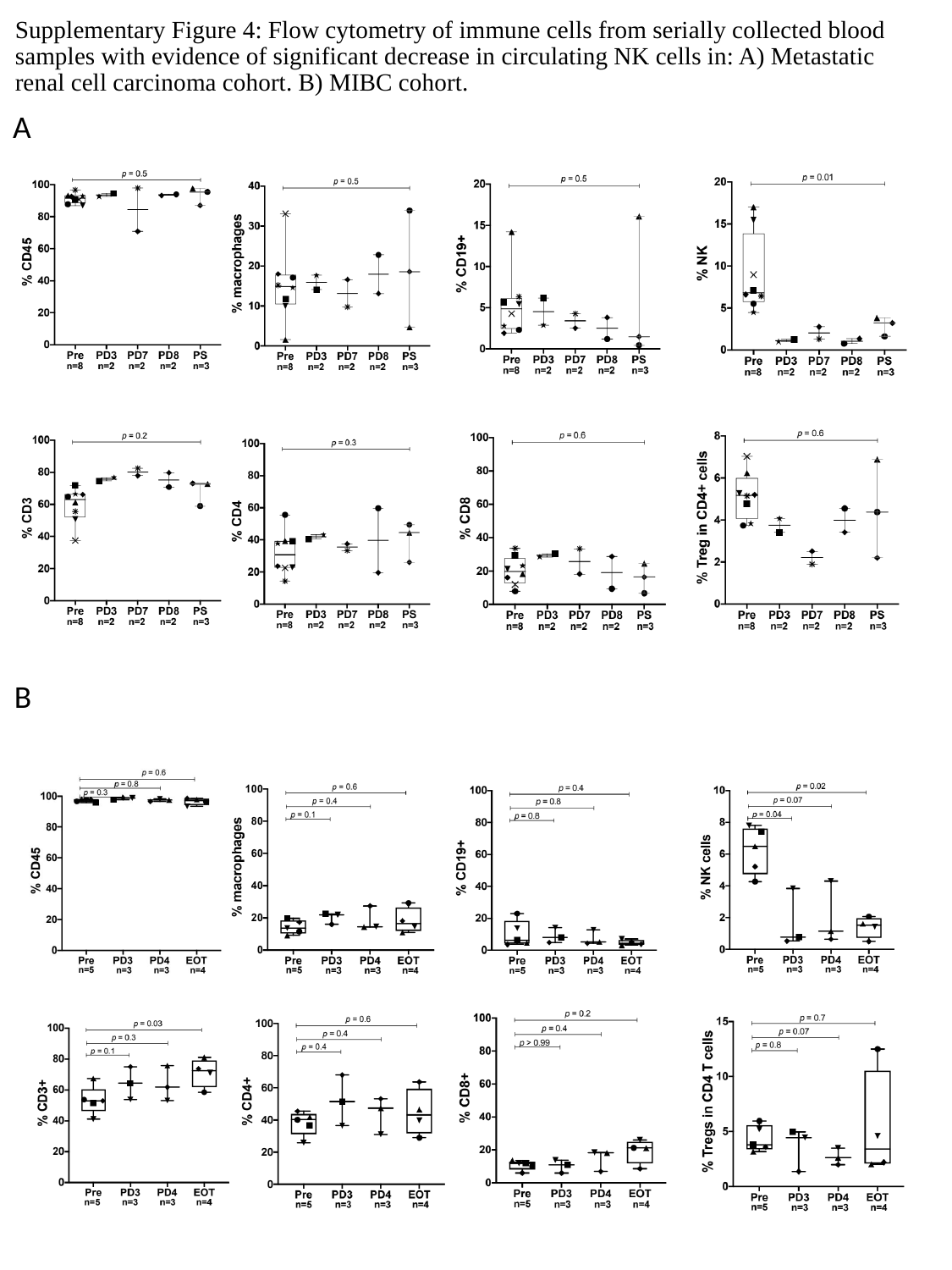

# Supplementary Figure 4: Flow cytometry of immune cells from serially collected blood samples with evidence of significant decrease in circulating NK cells in: A) Metastatic renal cell carcinoma cohort. B) MIBC cohort.
A
B
